# Reducing hospital admissions in older care home residents: a 4-year evaluation of the care home innovation Programme (CHIP)

**DOI:** 10.1186/s12913-020-4945-9

**Published:** 2020-02-06

**Authors:** Clarissa Giebel, Debbie Harvey, Asangaedem Akpan, Peter Chamberlain

**Affiliations:** 1 Institute of Population Health Sciences, University of Liverpool, Waterhouse Building B Block, Brownlow Street, Liverpool, L69 3GL England; 2 NIHR ARC NWC, Liverpool, England; 3 South Sefton Clinical Commissioning Group, Liverpool, England; 4 Liverpool University Hospitals NHS FT, Liverpool, England; 5 Institute of Ageing & Chronic Disease, University of Liverpool, Liverpool, England

**Keywords:** Care homes, System change, Training, Hospital attendance

## Abstract

**Background:**

Older care home residents frequently attend emergency departments with a high conversion to admissions. For this purpose, a novel Care Home Innovation Programme (CHIP) was introduced with the aim of reducing potentially avoidable hospital admissions by 30%. The aim of this study is to evaluate the implementation of this innovative service in practice.

**Methods:**

A total of 32 care homes with 1314 beds in South Sefton, Merseyside were invited to sign up to CHIP which was launched in April 2015 and continued in its entirety until June 2018. As part of the CHIP, care home matrons were introduced, new protocols were developed to address common presentations, a 24-h 7–day a week televideo system installed across all homes, and a quarterly training collaborative brought care homes together to learn and share good practices together. Data on emergency calls and calls resulting in conveyances were recorded over a four-year period, and analysed using frequency analysis.

**Results:**

In comparison to the 12 months prior to launch, over a four-year period, implementation of the CHIP resulted in a 15% reduction of emergency calls, and in a 19% reduction of conveyances to hospital.

**Conclusions:**

The South Sefton CHIP demonstrated itself an effective programme in reducing conveyances and consequently, hospital admissions of care home residents. This model will be superseded by the enhanced health in care homes being promoted by the NHS Long Term Care Plan.

## Background

When caring for someone in their own home becomes too stressful and demanding as a consequence of physical or mental ill health, people often move into a care home. In England and Wales, over 291,000 people aged 65 and above were residing in a care home in the last national count [11], representing 3.2% of older adults overall. The majority of care home residents are female, although there is a trend with an increase in male care home residents compared to before [11].

People move into a care home for different reasons, such as difficulties with everyday activities or behavioural problems [1, 14]. Due to the age of care home residents, they usually have multiple chronic conditions, including dementia, and have high levels of frailty [7]. Residents may deteriorate and require additional interventions from their general practitioner, community based staff, ambulance service and/or local hospital. There are cases of potentially avoidable hospital attendances [2, 6], which could be addressed by improved community services including an outreach service from a local hospital especially for cases where people are too frail to make the journey themselves. Hospitalisation can result in increased problems with everyday activities and mobility in older adults [4, 5], and adverse events can occur during the transition from hospital to discharge destinations [10]. This is particularly important when considering the care home population at large, who are mostly frail and dependent [8]. By attending a hospital, residents can get confused and return to the care home often worse than before. Therefore, hospitalisation and attending emergency departments should be avoided as much as possible, and care home residents should be supported and cared for safely in their residence.

Whilst training care home staff is frequently reported (i.e. [9]), it appears that only one previous intervention has investigated the effects of providing in-house care home support to avoid potentially avoidable Accident & Emergency (A&E) attendance [12]. By providing a quality improvement intervention, involving strategies to support care home staff identify problems early and communicate these, the intervention was effective in reducing A&E attendance in care home residents by 17% across 25 care homes. This multi-component quality improvement intervention included leadership and nursing home staff education, as well as collaborative telephone conference calls, and early warning tools, all targeted at improved identification, assessment, and management of health issues which could be dealt with by care home staff as opposed to calling an ambulance. However, with this intervention conducted in the United States under the Medicare structure, it remains to be seen whether this type of quality improvement intervention would be effective in other countries with different clinical roles, health care and care home systems in place.

The aim of this evaluation was to establish whether the novel Care Home Innovation Programme (CHIP), a quality improvement intervention, was effective in reducing potentially avoidable ambulance conveyances of older care home residents to hospitals by 30%, via holistic care by a multi-disciplinary team (MDT). This directly addresses one of the main priorities of the recently released NHS Long Term Plan (2019) to support people to age well, by ‘developing more rapid community response teams to prevent unnecessary hospital spells’ and by ‘upgrading NHS staff support to people living in care homes.

## Methods

### The care home innovation Programme (CHIP)

The CHIP was developed by a local Clinical Commissioning Group in the North West Coast area of England in order to provide a comprehensive support package to care home residents in the South Sefton region. The programme was set up as a result of finding high numbers of older care home residents with frailty having frequent hospital admissions in their final years of life. The programme was based on quality improvement principles with the inclusion of Plan Do Study Act (PDSA) cycles. As part of the CHIP, community matrons (CM) had a number of care homes allocated to them. A community care home matron is usually a senior nurse who may have a masters degree and non-medical prescribing qualifications. The components of the intervention included the CM proactively reviewing residents and supporting primary care to put in place an advance care plan, deal with acute minor illnesses, and a televideo system, which allowed care home staff to seek clinical advice for their residents especially after 5 pm and on weekends and bank holidays. In addition CHIP involved a more coordinated multi-disciplinary team approach to care home residents care, including district nurses, palliative care nurses, urgent care teams, community geriatricians, and medicines management. All of those involved in CHIP were involved in bi-monthly work stream meetings to encourage and optimise collaborative working. Table 1 outlines all elements of the CHIP. In the UK, 999 is a universal emergency call number and 111 is used for non-emergency medical help. Within the care homes, all 999 protocols were removed and replaced with evidence-based protocols for 13 specific situations including falls, head injuries and hydration. The clinical multi-disciplinary team provided training to care home staff to be able to make use of the specific protocols. All care homes were invited to quarterly collaboratives where education was provided along with support for quality improvement initiatives and CHIP updates. It is clear that these fora were critical for team building and joined up working for a shared vision. Finally care home carers were invited to a one-off clinical training program administered through a local university focussing on basic clinical assessment and application of protocols.

**Table 1:** Elements of the Care Home Improvement Programme

CHIP Element	Description
Community Matron	Senior nurses providing a weekday 9–5 service both reactive care for urgent presentations and care planning of patients resulting in an advanced care plan. A community matron. A community care home matron is usually a senior nurse who may have a masters degree and non-medical prescribing qualifications.
Televideo remote advanced nurse practitioner	Each care home that agreed to participate had a laptop with webcam supplied and installed free of charge. This provided 24-h access to a band 7 nurse in Airedale NHS Trust who could provide video assessment
General Practitioner	Provide support and advice as the registered doctor
Community Geriatrician	Provide support and advice including joint visits or reviews
CHIP protocols	13 clinically derived protocols that follow expert guidance on the initial management of common presentation e.g. falls, head injury, shortness of breath etc.
Training to care home staff	Basic training package for healthcare assistants in taking observations and applying protocols provided by Edge Hill University
Newsletter	Monthly newsletter
Quality Improvement Collaborative meetings	Quarterly meetings allowing care homes to be trained, update on progress, introduced to services and share good practice

### Dataset

Data were collected from April 2014 to April 2019. Data included information on total 999 calls from care homes, number of conveyances, calls to the televideo hub, and General Practitioner out-of-hours care home visits. Total data on 999 calls was generated from standard ambulance data service monitoring data. No transcripts are available within this data set. When a patient rings 999, they do not have to provide consent to allow the emergency services to intervene as they have provided implicit consent by ringing in the first place. However, the emergency services are required to collate anonymised activity data as part of their contract. This is taken from the activity data key performance indicators. All data sources were collated from key performance indicators as part of contracts held with respective provider organisations, and are thus from standardised administrative source, as supplied to the Clinical Commissioning Group by the respective provider. As such, we can be confident in the data sources as these are generated as part of the respective organisations fundamental activities for delivery and contractual obligation. No ethical approval was required for this study, as this was a secondary data analysis of an implemented service, and only anonymised systems data were collected with no individual care home residents having participated as such in this study. For this first evaluation of the CHIP model, 32 care homes were invited and participated to varying degrees in the programme.

### Data analysis

Data were analysed using time series analysis using run charts with correlation and Mann-Whitney non-parametric tests run through QI Macros. Shifts in the median represent 6 data points under the baseline as per run chart rules [13] resulting in a re-calculation of the median for the respective data.

## Results

**Fig. 1:**
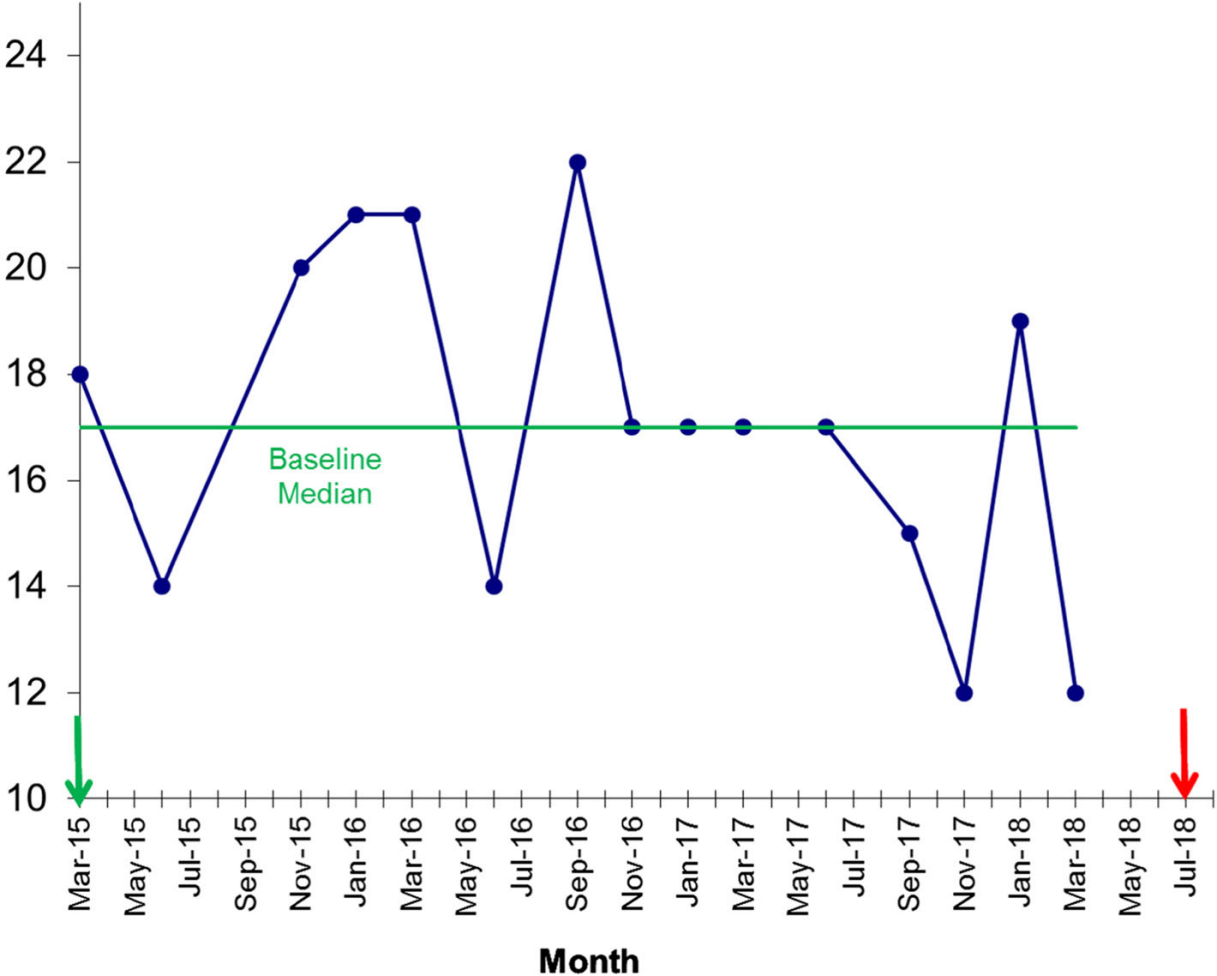
Run chart showing attendance at quality improvement collaboratives over the course of the program. Arrows indicate start and end date of the programme and green line baseline median. Total number of care homes invited = 32

**Fig. 2:**
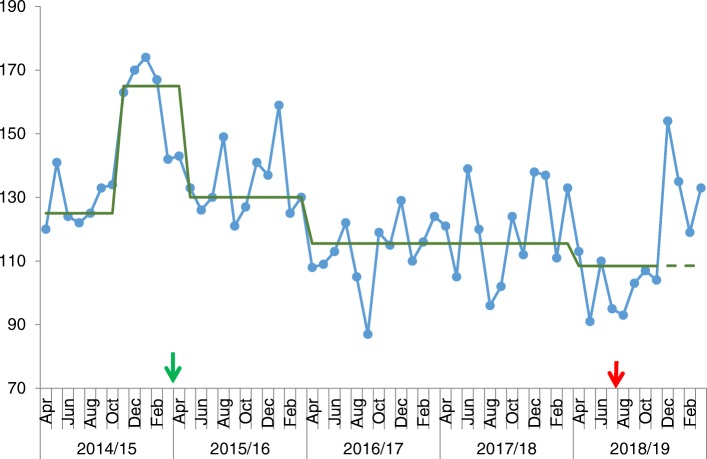
Run chart showing 999 calls from South Sefton Care Homes. Legend: X-axis shows time of the project, and the y-axis shows the number of 999 calls (per month). Arrows indicate start and end date of the programme. Green line indicates baseline median

A total of 32 care homes were invited to be involved in CHIP and all participated. This represented 17 residential homes and 15 nursing homes. The baseline standard of these homes was “good” in 56%, “required improvement” in 44%, and one home was graded “inadequate” in the national UK Care Quality Commission regulatory inspection. Of these, two thirds regularly attended the training collaborative with a median attendance of 17/32 (see Fig. 1). In the 12 months prior to CHIP, the mean number of 999 calls across the 32 care homes was 143 per month (see Fig. 2). Time-series analysis showed that the number of 999 calls made was reduced by 15.1% over the three-year period compared to the 12 months prior to CHIP (*p* = 0.002, Mann-Whitney). Analysis using run-chart rules demonstrates a shift from the baseline median in April 2016 as the program gained momentum. In addition, there is a loss of seasonal variation seen prior to CHIP. Nine hundred ninety-nine calls start to rise after the program ends mid-2018 further validating the impact of the program.

**Fig. 3:**
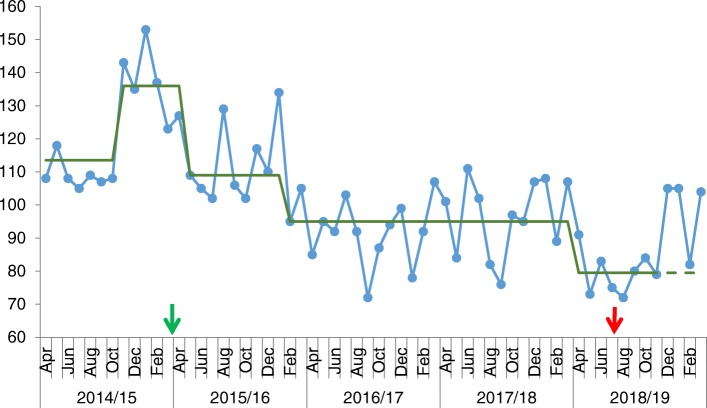
Run chart showing conveyances to hospital from South Sefton Care Homes following 999 call. Legend: X-axis shows time of the project, and the y-axis shows the number of conveyances to hospital (per month). Arrows indicate start and end date of the programme. Green line indicates baseline median

**Fig. 4:**
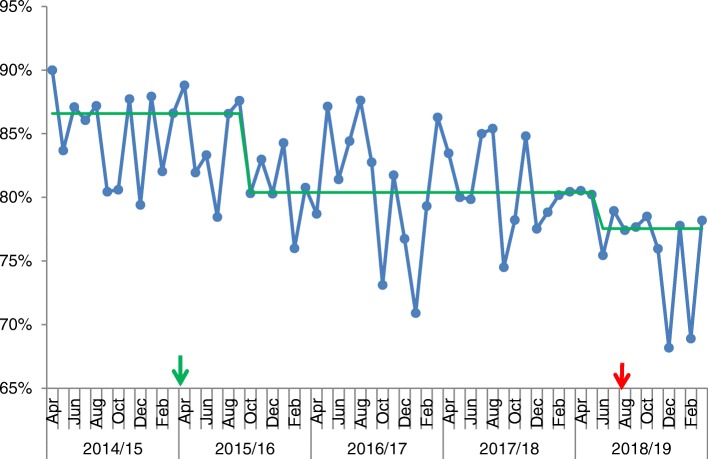
Run chart showing % of 999 Calls from South Sefton Care Homes which Resulted in a Conveyance. Legend: X-axis shows time of the project, and the y-axis shows the percentage of 999 calls (per month). Arrows indicate start and end date of the programme. Green line indicates baseline median

Given the complexity and frailty of the cohort it can be assumed that virtually all conveyances to hospital for urgent care are via the paramedic ambulance. Over the three-year period, time-series analysis showed an average 18.7% reduction in calls resulting in hospital conveyances compared to 12 month baseline (see Figs. 3 and 4). As per the call frequency, there is a loss of seasonal variation seen prior to CHIP.

**Fig. 5:**
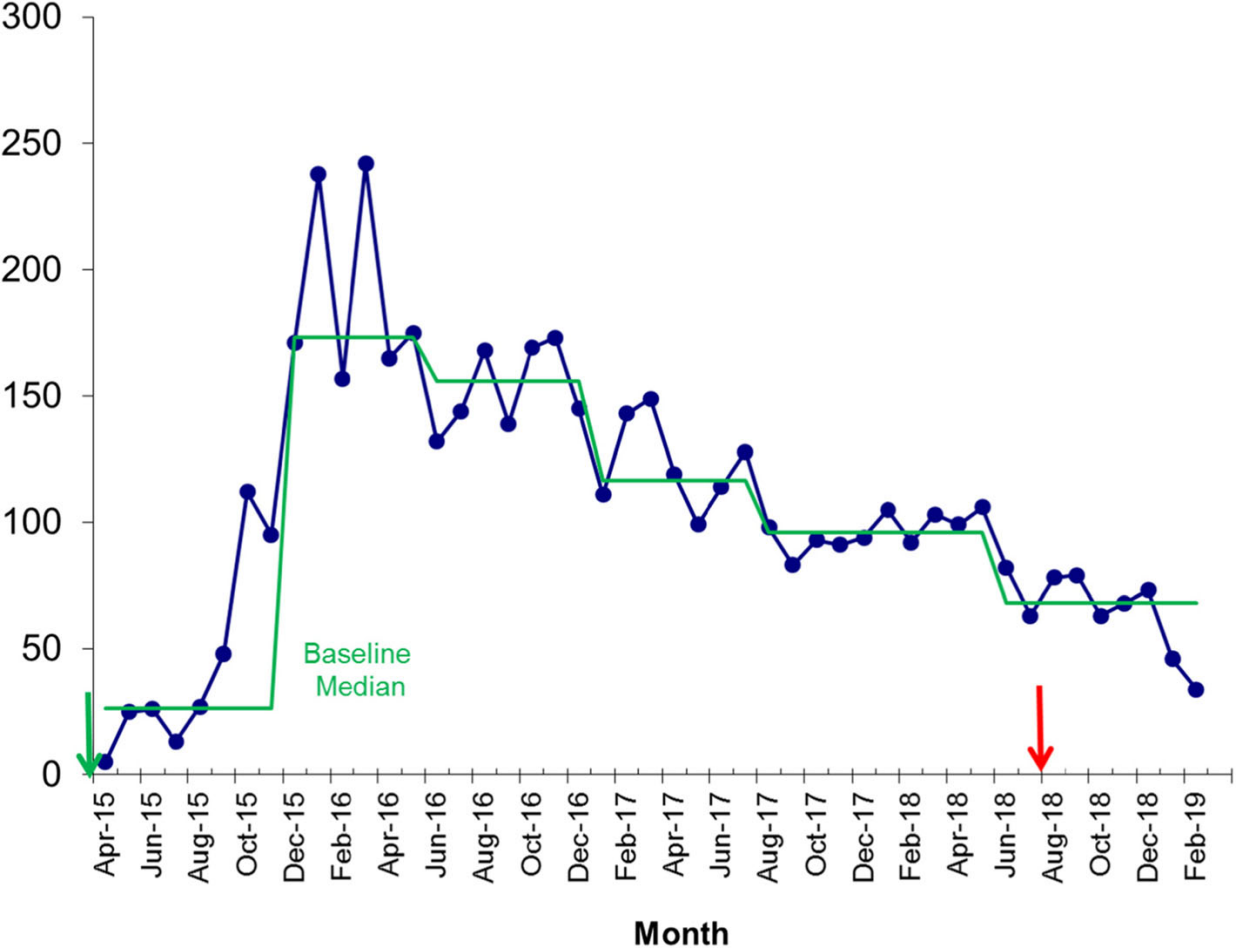
Run chart showing number of calls to televideo hub per month from South Sefton Care Homes. Legend: X-axis shows time of the project, and the y-axis shows the number of calls to a televideo hub (per month). Arrows indicate start and end date of the programme and green line baseline median

Scatter diagram between televideo calls and ambulance conveyances showed a weak inverse correlation but with a R^2^ of only 0.13. There was a similar pattern between televideo calls and GP out-of-hours visits with R^2^ of 0.21. Figure 5 shows the number of televideo calls over the programme period.

## Discussion

This is one of the first studies showing an effective implementation of a service to reduce potentially avoidable hospital admissions in care home residents. The CHIP service effectively reduced hospital admissions by 19%, by employing a MDT, a televideo system, and replacing all previous incident protocols with new ones and training up care home staff to employ the new system. System improvements can be very difficult to achieve due to the number of factors involved that need to be changed including staff training, alternative to 999, senior clinical input etc.

The time series data supports the impact of the interventions with a shift in the data and loss of seasonal variation. There is an early rise in both 999 calls and conveyances following the cessation of the program mid-2018. A 7% shift in the proportion of patients conveyed to hospital following 999 call is likely to represent the impact of the care planning aspect of the program alongside cultural changes within the ambulance provider.

To our knowledge, only one previous study has explored the implementation of a similar service in the USA [12], with current findings corroborating the benefits of a care home training programme to reduce potentially avoidable hospital attendances in a UK setting. Over a 6-month period, and covering 25 care homes, Ouslander et al.’ [12] service resulted in a 17% reduction in hospital attendance. With varying healthcare systems between both countries, and a different, even more holistic approach in the CHIP, this study adds important evidence showing that this type of service can be successful in the UK also.

One element that was considered difficult throughout the implementation of the service, was the high staff turnover including both managers and care staff [3]. As a result, new care home staff needed to be trained to learn how to use the new protocols and how to employ the televideo system as opposed to merely calling 999 if an incident occurred. Ongoing repetition was required to ensure consistency of approach. Other challenges included cultural differences between homes and lack of contractual levers, suggesting different factors that can hinder the effective implementation of the service.

The impact of televideo on conveyances is difficult to assess. Following an initial spike in use after implementation there was a gradual decline in use until the CHIP program was terminated. The feedback from care homes cited increasing difficulty getting through to the televideo provider leading to long waits and reversion to 111 leading to a rise in GP out-of-hour attendances. In addition, the process changed from initial nurse assessment to initial non-nurse assessment. Televideo was most likely being used mostly for minor presentations. As such it is likely that the greatest impact on conveyances was through the engagement in the training and quality improvement collaborative alongside the impact of the community matrons care planning and reactive care to urgent presentations. As matrons worked alongside the same homes, relational coordination was established, furthering trust and influence that enabled subsequent behavioural change within care homes.

### Limitations

Findings from this study need to be considered in light of some limitations. No individual patient data were collected as part of this evaluation, as the focus was set solely on systems-level approaches and the number of conveyances out of care homes. However, future evaluations of the CHIP programme would benefit from including patient characteristics and assessments, including the quality of life of care home residents by being supported by trained care home staff in the care home environment. Furthermore, this is a regional analysis of CHIP provided to care homes. However, with 32 care homes having been involved, findings from this study are representative of the North West Coast region of England, with each care home covering between 10 and 176 residents (an average of 38 residents per care home). With all 32 care homes participating in the CHIP intervention, no control sites were included. Therefore, the effects of the intervention were measured in terms of time analysis for across the care homes, and not by comparing care homes which had received the intervention with control sites. One home refused installation of televideo while three homes who did have it installed never used it. All homes engaged with care home matrons but cases per care home are not available. Future evaluations should include control sites to fully evaluate the CHIP intervention. This study also did not include any cost analysis, which should be considered in future evaluations of the intervention. Finally, external factors were not tracked in detail. There was no known major external change to the local system in relation to health providers, epidemics, or number of beds available that would account for these results.

## Conclusions

This four-year evaluation of the CHIP implementation has shown that CHIP was effective in reducing potentially avoidable hospital admissions in care home residents. Considering the frailty and vulnerability of care home residents [7], supporting residents to receive appropriate care in their own home setting (the care home) is important to prevent adverse events related to a hospital attendance and or admission. The next step will be to roll out components of the CHIP in other regions across the country, and internationally with potential adaptations to be country-specific. The CHIP is clearly significant in supporting care home residents with health issues in the comfort of their care home. This is validated by the recent inclusion of enhanced health in care homes which mirrors most of what CHIP did and can contribute to reducing high healthcare costs by training up care home staff to deal with frequently occurring health problems in the care home environment without unnecessary hospital visits.

## Data Availability

The data that support the findings of this study are available from Sefton CCG but restrictions apply to the availability of these data, which were used under license for the current study, and so are not publicly available. Data are however available from the authors upon reasonable request and with permission of Sefton CCG.

## Abbreviations

A&E: Accident & Emergency
CHIP: Care Home Innovation Programme
MDT: Multidisciplinary team
NHS: National Health Service
PDSA: Plan Do Study Act
